# Network-based approach to identify biomarkers predicting response and prognosis for HER2-negative breast cancer treatment with taxane-anthracycline neoadjuvant chemotherapy

**DOI:** 10.7717/peerj.7515

**Published:** 2019-09-03

**Authors:** Cui Jiang, Shuo Wu, Lei Jiang, Zhichao Gao, Xiaorui Li, Yangyang Duan, Na Li, Tao Sun

**Affiliations:** 1 Department of Medical Oncology, Cancer Hospital of China Medical University, Liaoning Cancer Hospital and Institute, Shenyang, Liaoning, China; 2 Institute of Translational Medicine, China Medical University, Shenyang, Liaoning, China

**Keywords:** Response, Breast cancer, Neoadjuvant chemotherapy, Prognosis, WGCNA

## Abstract

**Objective:**

This study aims to identify effective gene networks and biomarkers to predict response and prognosis for HER2-negative breast cancer patients who received sequential taxane-anthracycline neoadjuvant chemotherapy.

**Materials and Methods:**

Transcriptome data of training dataset including 310 HER2-negative breast cancer who received taxane-anthracycline treatment and an independent validation set with 198 samples were analyzed by weighted gene co-expression network analysis (WGCNA) approach in R language. Gene ontology (GO) terms and Kyoto Encyclopedia of Genes and Genomes (KEGG) pathways analysis were performed for the selected genes. Module-clinical trait relationships were analyzed to explore the genes and pathways that associated with clinicopathological parameters. Log-rank tests and COX regression were used to identify the prognosis-related genes.

**Results:**

We found a significant correlation of an expression module with distant relapse–free survival (HR = 0.213, 95% CI [0.131–0.347], *P* = 4.80E−9). This blue module contained genes enriched in biological process of hormone levels regulation, reproductive system, response to estradiol, cell growth and mammary gland development as well as pathways including estrogen, apelin, cAMP, the PPAR signaling pathway and fatty acid metabolism. From this module, we further screened and validated six hub genes (CA12, FOXA1, MLPH, XBP1, GATA3 and MAGED2), the expression of which were significantly associated with both better chemotherapeutic response and favorable survival for BC patients.

**Conclusion:**

We used WGCNA approach to reveal a gene network that regulate HER2-negative breast cancer treatment with taxane-anthracycline neoadjuvant chemotherapy, which enriched in pathways of estrogen signaling, apelin signaling, cAMP signaling, the PPAR signaling pathway and fatty acid metabolism. In addition, genes of CA12, FOXA1, MLPH, XBP1, GATA3 and MAGED2 might serve as novel biomarkers predicting chemotherapeutic response and prognosis for HER2-negative breast cancer.

## Introduction

Breast cancer (BC) remains the second most frequently occurred cancer worldwide as well as the most common cancer in women (Siegel, Miller & Jemal, 2017). At present, breast cancer is the primary cause of death in women all over the world and becomes one of the most expensive tumors to treat (Harbeck & Gnant, 2017). Until now, three key protein biomarkers have shown great help in guiding prognosis and therapy for BC, including progesterone receptor (PR), estrogen receptor (ER), and human epidermal growth factor (EGF) receptor 2 (HER2) (De la Mare et al., 2014). Breast cancers with no expression of these three markers are generally classified as triple negative breast cancers (TNBCs) (Abramson et al., 2015).

In recent years, neoadjuvant chemotherapy has emerged as an increasingly critical approach in the systemic treatment of women with breast cancer (Rapoport et al., 2014). Systemic neoadjuvant chemotherapy is widely utilized along with surgery and radiotherapy for the management of patients with locally advanced BC (Read et al., 2015; Teshome & Hunt, 2014; Untch et al., 2014). The HER (human epidermal growth factor receptor) family represents a series of structurally associated receptor tyrosine kinases controlling the growth and development of multiple organs including the breast (Nuciforo et al., 2015). HER2 has been reported to cause aggressive behaviors of cancer cells including rapid growth and frequent metastasis (Wieduwilt & Moasser, 2008). Targeting HER2 in BC has shown effectiveness in clinical trial, which offers a reliable treatment option (Duffy et al., 2015; Krishnamurti & Silverman, 2014; Moasser & Krop, 2015).

Currently, chemotherapy is commonly adopted in HER2-negative breast cancer management, of which taxane-anthracycline combination regimens have been regarded as typical neoadjuvant chemotherapeutic strategies (Hanusch et al., 2015). Taxanes represent a series of drugs used in the treatment of cancer including paclitaxel and docetaxel, which affect microtubules structures of cancer cells to block their division (Ghersi et al., 2015; Murray et al., 2012). Anthracyclines such as doxorubicin and epirubicin induce DNA intercalation and lead to apoptosis of breast cancer cells (Greene & Hennessy, 2015; Turner, Biganzoli & Di Leo, 2015). At present, no clinically useful prognostic or predictive examination for patients with HER2 breast cancer have been established.

Although recent improvements of the chemotherapy, hormone therapy, radiotherapy and immune therapy have greatly benefit the prognosis for BC patients, obvious individual differences are observed in the outcomes of BC treatments on account of heterogeneity. As a result, novel and robust biomarkers are urgently required to predict the chemotherapy sensitivity and survival for HER2-negative BC patients. In this study, by means of weighted gene co-expression network analysis (WGCNA) (Langfelder & Horvath, 2008), we systematically analysed microarray-based gene expression profiling data of 310 HER2-negative BC cases treated with taxane-anthracycline neoadjuvant chemotherapy. In addition, another independent validation set with 198 BC samples were also analysed in order to identify new biomarker to predict response and prognosis for HER2-negative BC patients who received sequential taxane-anthracycline neoadjuvant chemotherapy.

## Materials and Methods

### Analyzed datasets

The training dataset adopted for network analysis contained 310 HER2-negative breast cancer samples, all of which had conducted taxane-anthracycline treatment. All the data were obtained at GEO database with accession number of GSE25055. Altogether 198 samples was independently analyzed to validate the relation of gene modules/hub genes with survival of HER2-negative BC patients treated with taxane-anthracycline. Before data analysis, batch effect was removed using the removeBatchEffect function in limma package (Ritchie et al., 2015). The Data Normalization was performed by the RMA function in limma function. These samples were downloaded from GEO with the accession numbers GSE25065. Chemoresistance included extensive residual cancer burden (RCB) or early relapse, while chemo-sensitivity represented pathologic complete response (pCR) or minimal RCB. As we focused on HER2-negative BC, we removed the HER2-positive patients. All the samples were hybridized using Affymetrix Human Genome U133A Array according to standard Affymetrix protocols. Original gene expression counts were analyzed through robust multiarray average algorithms. Because genes with limited variation in expression often mean noise, we only selected relatively variant genes for construction of network. The variabilities of genes were assessed by median absolute deviation (MAD) (Chen et al., 2018).

### Construction of gene co-expression network

We then constructed the gene co-expression network using the WGCNA package by R (Langfelder & Horvath, 2008). Power values were filtered out through means of WGCNA in constructing the co-expression modules. Scale independence and average connectivity assessment of modules holding diverse power value were conducted via gradient analysis. Proper power value was selected when the scale independence value comes to 0.9. WGCNA method was then adopted to construct the co-expression network and obtain the gene information in the most relevant module. We performed Heatmap by R language to illustrate the strength of the association between different modules. As a representative of the gene expression profiles of a module, module eigengene (ME) was used to evaluate the relationship between module and distant relapse–free survival (DRFS).

### Identification clinical traits-related modules

After we built the gene expression related module, the module–trait relationship (MTR) analysis was used to analyze the relation between the module and clinical traits (Langfelder & Horvath, 2008). Pearson’s correlation test was used to explore the association of MEs with clinical traits such as sensitivity, stage, pam50 and grade. We also calculated module preservation via modulePreservation function in order to assess if the module is stable and repeatable through datasets. Zsummary of preservation statistics represent the stability of certain statistical analysis. Zsummary value over 10 strongly indicated preserved and robust module (Langfelder et al., 2011). Negative correlation between Preservation statistics medianRank with module preservation were observed. The protein–protein interaction network of module genes was performed by STRING (https://string-db.org).

### Survival analysis

We used the ‘survival’ package in R to fulfill the survival analysis. The Hazard Ratio as well as 95% CI were calculated by Cox regression model. We generated the curve for survival through Kaplan–Meier method. Individual ME was classified as higher and lower expression by median value to perform multigene associations.

### Gene ontology and pathway Enrichment analysis

In order to explore the possible biological functions and pathways enriched by genes within the module, the clusterprofiler package of R was adopted to demonstrate gene ontology items (Ashburner et al., 2000) and Kyoto Encyclopedia of Genes and Genomes (KEGG) pathways (Kanehisa et al., 2004).

### Identification of hub genes

We then selected the top 10 genes in the DRFS-associated module as the candidate hub DRFS-associated genes by the *p* value of their prognosis analysis. Then we verified the genes in the validation set. We then analyzed the association between hub genes and taxane-anthracycline response.

## Results

### Classification of breast cancer subtypes

As was shown in Table 1, the training dataset contained 310 samples while the validating dataset included 198 samples. Sensitive numbers of taxane-anthracycline treatment in training and validating sets accounted for 36.5% and 28.3%, respectively. Samples were included within subtypes according to classification based on the above classifiers for the following analyses.

**Table 1:** Basic characteristics of the datasets.

**Category**	**Training dataset**	**Validating dataset**
N	310	198
Age (mean (sd))	50.17 (10.39)	49.24 (10.57)
Event = 1 (%)	66 (21.3)	45 (22.7)
Time_years (mean (sd))	2.82 (1.69)	3.22 (1.50)
Type (%)		
Basal	122 (39.4)	67 (33.8)
Her2	20 (6.5)	17 (8.6)
LumA	99 (31.9)	61 (30.8)
LumB	44 (14.2)	34 (17.2)
Normal	25 (8.1)	19 (9.6)
Sensitive (%)	113 (36.5)	56 (28.3)
Stage (%)		
I	24 (7.7)	27 (13.6)
II	165 (53.2)	63 (31.8)
III	121 (39.0)	108 (54.5)
Grade (%)		
I	27 (8.7)	11 (5.6)
II	117 (37.7)	107 (54.0)
III	151 (48.7)	80 (40.4)
IV	15 (4.8)	0(0)

**Figure 1:**
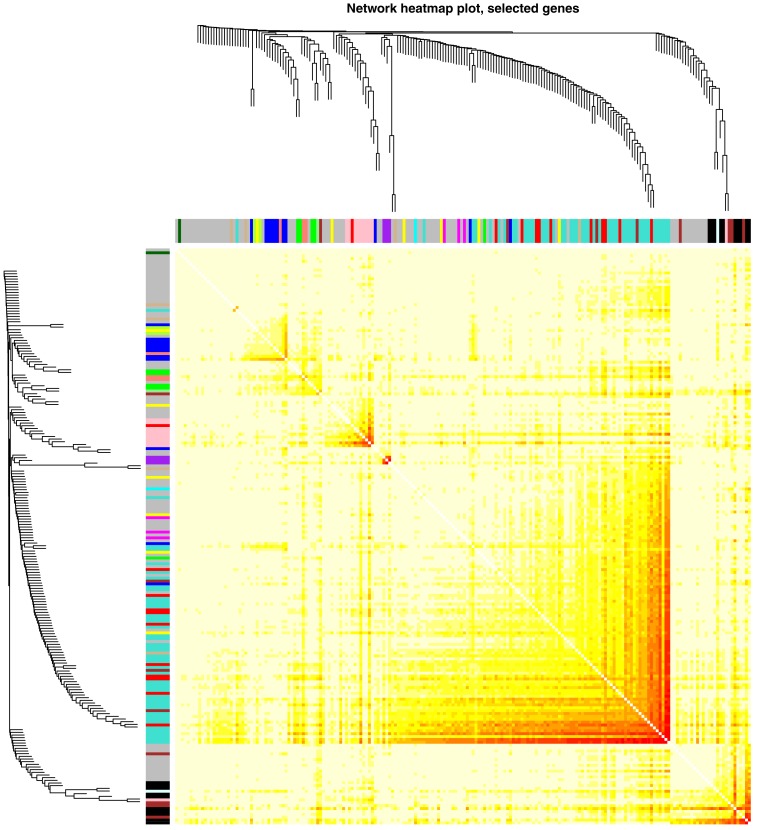
Network heatmap plot representing the interaction relationship analysis of co-expression genes for HER2-negative breast cancer patients.

### Gene co-expression network of breast cancer

Altogether 5,571 most variant genes were selected according to MAD in order to perform additional analysis. The connectivity among genes was a scale-free network distribution if the value of soft thresholding power β equals to 5 (Fig. S1). Altogether 10 modules were filtered via hierarchical clustering as well as Dynamic branch Cutting. The module was given an individual color as identifiers. Interaction relationship analysis of co-expression genes was shown in Fig. 1. Gene numbers within modules ranged from 35 to 724. If the gene set belong to no module, this was the grey module. Threshold selection of WGCNA analysis was shown in Fig. S1.

### Module–clinical trait correlations and preservation

**Figure 2:**
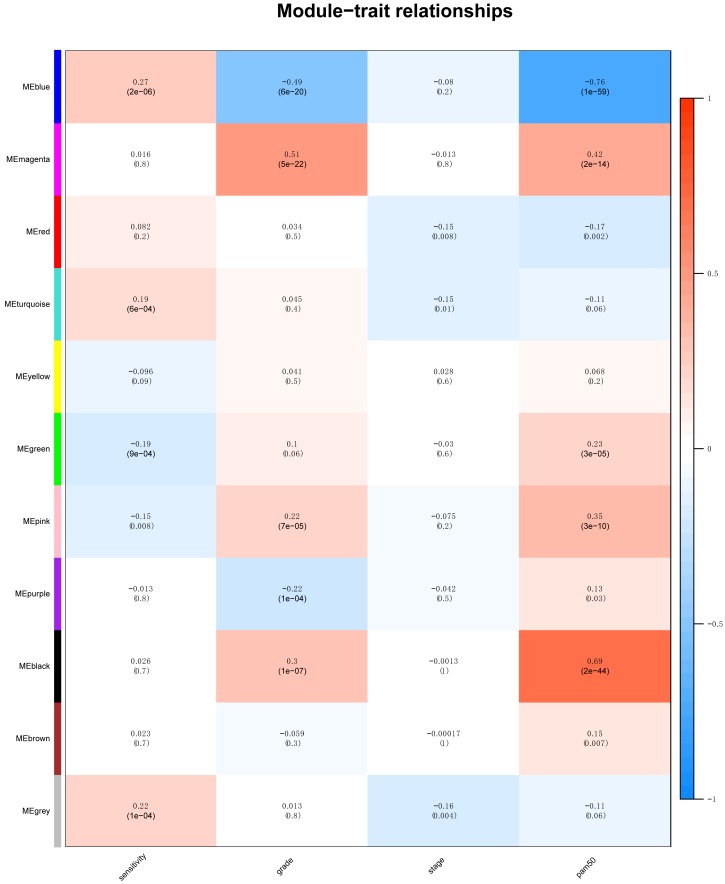
The module–clinical trait relationships of genes involved in clinicopathological parameters (sensitivity, grade, stage and pam50) of HER2-negative breast cancer patients.

**Figure 3:**
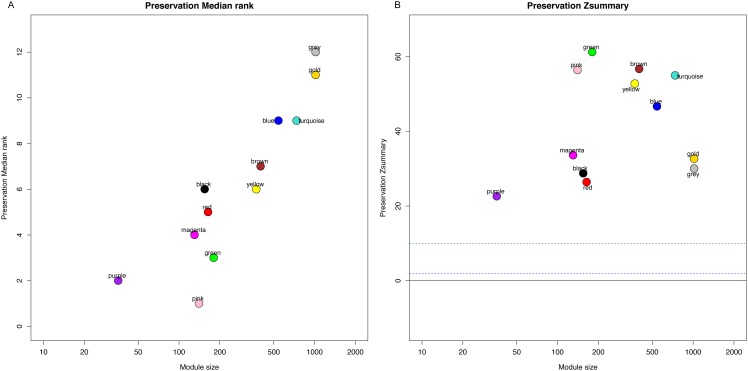
Module preservation analysis in the validating set to represent the stability and robustness of module analysis. (A) Preservation Median rank, (B) Preservation Zsummary.

**Figure 4:**
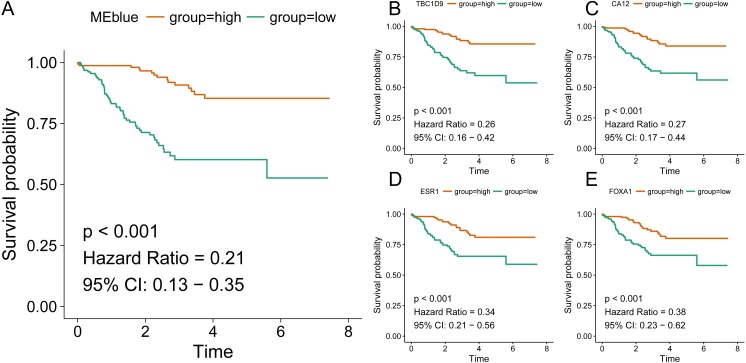
Association of blue module and top hub genes with survival for HER2-negative breast cancer patients. A, blue module; B, TBC1D9 gene; C, CA12 gene; D, ESR1 gene; E, FOXA1 gene.

Identification of clinical trait related genes is of great interest to elucidate the underlying mechanisms behind the clinical trait. In our study, the clinical parameters of breast cancer patients, including sensitivity, grade, stage and pam50 classifiers were involved in MTR analysis. As was suggested in Fig. 2, sensitivity, grade and pam50 were associated with blue module (*r* = 0.27, *P* = 2e−; *r* =  − 0.49, *P* = 6*E* − 20; and *r* =  − 0.76, *P* = 1E-59). Stage was associated with red module (*r* =  − 0.15, *P* = 0.008). Then we performed module preservation analysis in validating set. As was shown in Fig. 3, all of the modules’ zsummery statistics were greater than 10. Finally, significant relation of module blue MEs (HR = 0.213, 95% CI = 0.131–0.347, *P* = 4.80E−09) (Table 2 and Fig. 4A) with DRFS was identified. As the module blue was also associated with sensitivity, grade, stage and pam50, we selected module blue as the hub module.

### Enrichment analysis of the blue module

**Figure 5:**
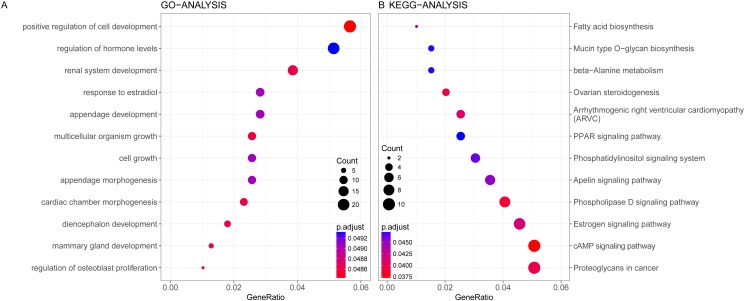
Gene Ontology analysis (A) and KEGG pathway enrichment analysis (B) for genes in the prognosis-related blue module.

GO and KEGG enrichment analysis were conducted on the genes in blue module. Altogether 163 terms showed differences in GO enrichment (Table 3). As was illustrated in Fig. 5, this module was related with regulation of hormone levels, reproductive system development, response to estradiol, cell growth and mammary gland development according to GO analysis. As for KEGG analysis, 51 pathways were associated with blue module including Estrogen signaling pathway, Apelin signaling pathway, cAMP signaling pathway, PPAR signaling pathway and fatty acid metabolism. The protein–protein interaction network of genes in blue module was shown in Fig. S2.

### Identification of hub genes associated with survival

Hub genes tend to exert core functions in a closely related network. Of the 532 genes within blue module, we selected the ten most relevant genes as the candidate hub genes (TBC1D9 (*r* = 0.902), CA12 (*r* = 0.899), ESR1 (*r* = 0.884), FOXA1 (*r* = 0.875), MLPH (*r* = 0.873), XBP1 (*r* = 0.867), AGR2 (*r* = 0.865), GATA3 (*r* = 0.860), SLC39A6 (*r* = 0.846), and MAGED2 (*r* = 0.813)). As was shown in Figs. 4B–4E and Table 4, all of the ten genes were significantly associated with DRFS. The prognosis results of these ten genes adjusted for stage and grade also indicated significance, which were listed in Table S1.

**Table 2:** Prognosis analysis of WGCNA module.

**Module**	**Gene count**	**HR**	**95% CI**	***p*-value**
Black	152	1.778	1.096-2.884	0.02
Blue	532	0.213	0.131-0.347	<0.001
Brown	393	1.231	0.760-1.994	0.399
Green	177	1.2	0.740-1.944	0.459
Magenta	128	1.897	1.171-3.075	0.011
Pink	138	1.779	1.098-2.884	0.021
Purple	35	1.041	0.642-1.686	0.871
Red	161	0.705	0.434-1.144	0.153
Urquoise	724	0.621	0.381-1.011	0.048
Yellow	365	0.871	0.538-1.411	0.575

**Table 3:** Enrichment analysis of blue module.

**Ontology**	**ID**	**Description**	**Gene ratio**	**adjusted *P***	**Count**
BP	GO:0010817	Regulation of hormone levels	27/389	0.002395409	27
BP	GO:0061458	Reproductive system development	28/389	0.000408073	28
BP	GO:0032355	Response to estradiol	12/389	0.004058761	12
BP	GO:0016049	Cell growth	26/389	0.004761362	26
BP	GO:0030879	Mammary gland development	11/389	0.013827815	11
CC	GO:0043025	Neuronal cell body	30/401	1.00209E-05	30
CC	GO:0044297	Cell body	30/401	9.79994E-05	30
CC	GO:0031252	Cell leading edge	20/401	0.020992643	20
CC	GO:0030315	T-tubule	6/401	0.027790409	6
CC	GO:0045177	Apical part of cell	19/401	0.027790409	19
MF	GO:0015267	Channel activity	25/387	0.01758155	25
MF	GO:0022803	Passive transmembrane transporter activity	25/387	0.01758155	25
MF	GO:0005216	Ion channel activity	22/387	0.042958334	22
MF	GO:0005261	Cation channel activity	18/387	0.042958334	18
MF	GO:0022838	Substrate-specific channel activity	22/387	0.043597986	22
KEGG	hsa04915	Estrogen signaling pathway	14/197	1.35763E-05	14
KEGG	hsa04371	Apelin signaling pathway	11/197	0.000946647	11
KEGG	hsa04024	cAMP signaling pathway	13/197	0.002153221	13
KEGG	hsa03320	PPAR signaling pathway.	7/197	0.003222631	7
KEGG	hsa01212	Fatty acid metabolism	5/197	0.008276217	5

**Table 4:** Association relationship between hub genes with survival.

	**Training dataset**			**Validating dataset**		
**Gene**	**HR**	**95% CI**	***p***	**HR**	**95% CI**	***p***
TBC1D9	0.258	0.159–0.420	3.38E−07	0.238	0.132–0.427	1.26E−05
CA12	0.272	0.167–0.442	6.32E−07	0.271	0.151–0.488	5.26E−05
ESR1	0.343	0.211–0.557	2.56E−05	0.405	0.225–0.727	0.003657406
FOXA1	0.381	0.235–0.619	0.000139831	0.356	0.198–0.64	0.001033344
MLPH	0.343	0.211–0.558	2.62E−05	0.404	0.225–0.725	0.003540279
XBP1	0.250	0.154–0.407	1.63E−07	0.367	0.205–0.659	0.001487792
AGR2	0.381	0.234–0.618	0.000138311	0.452	0.252–0.812	0.009983604
GATA3	0.295	0.181–0.48	2.39E-06	0.203	0.113–0.366	2.02E−06
SLC39A6	0.297	0.183–0.482	2.68E−06	0.272	0.151–0.49	5.65E−05
MAGED2	0.302	0.186–0.492	2.68E−06	0.352	0.196–0.632	0.000874498

### Identification of hub genes involved in taxane-anthracycline resistance

**Figure 6:**
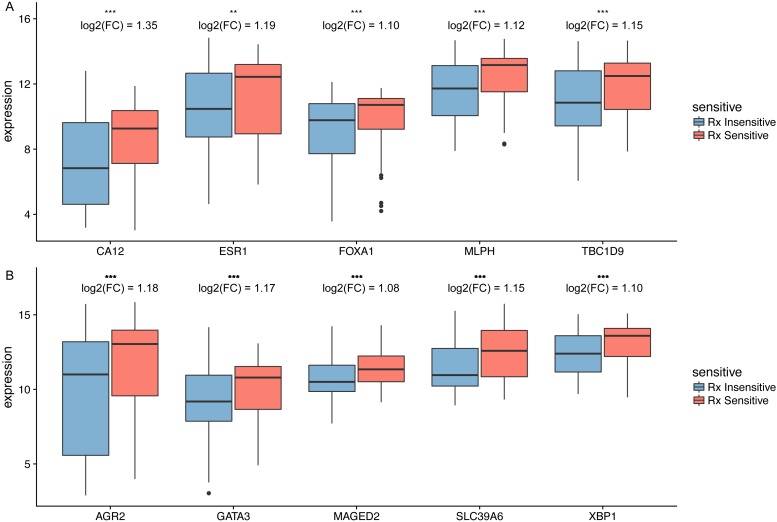
The differential expression of potential hub genes in the sensitive and insensitive group of HER2-negative breast cancer patients who received taxane-anthracycline neoadjuvant chemotherapy. (A) Top five hub genes involved in taxane-anthracycline resistance. (B) Top 6–10 hub genes involved in taxane-anthracycline resistance. The fold change (FC) of differential expression of potential hub genes were shown.

According to our findings, we suggested that increased expression of hub genes were associated with prolonged survival in HER2-negative BC patients treated with taxane-anthracycline, thus the identified hub genes may participate in taxane-anthracycline resistance. To validate this hypothesis, we analyse the relationship between hub gene expression and taxane-anthracycline sensitivity. In the training set, all the hub genes demonstrated significant difference between insensitive and sensitive group (Fig. 6). Moreover, in the validating dataset, six of ten genes (CA12, FOXA1, MLPH, XBP1, GATA3 and MAGED2) showed significant difference between two groups (Table 5).

**Table 5:** Association relationship between hub genes with taxane-anthracycline resistance.

			**Training set**		**Validing set**	
**Gene**	**Full name**	**Location**	**OR (95% CI)**	***p***	**OR (95% CI)**	***p***
TBC1D9	TBC1 domain family member 9	4q31.21	1.272(1.122–1.448)	0.0003663	1.113(0.957–1.31)	0.1635
CA12	Carbonic anhydrase 12	15q22.2	1.232(1.123–1.357)	2.40E-05	1.14(1.011–1.294)	0.02608
ESR1	Estrogen receptor 1	6q25.1-q25.2	1.17(1.055–1.302)	0.007904	1.163(0.999–1.361)	0.08573
FOXA1	Forkhead box A1	14q21.1	1.259(1.094–1.461)	0.0002293	1.3(1.053–1.641)	0.01121
MLPH	Melanophilin	2q37.3	1.321(1.145–1.535)	0.0001142	1.261(1.027–1.568)	0.01997
XBP1	X-box binding protein 1	22q12.1	1.501(1.256–1.809)	2.87E-06	1.253(1.001–1.599)	0.04864
AGR2	Anterior gradient 2, protein disulphide isomerase family member	7p21.1?	1.157(1.081–1.243)	1.20E-05	1.083(0.994–1.188)	0.08473
GATA3	GATA binding protein 3	10p14	1.26(1.119–1.427)	0.0001042	1.137(1.002–1.334)	0.03515
SLC39A6	Solute carrier family 39 member 6	18q12.2	1.399(1.216–1.617)	2.46E-06	1.167(0.976–1.4)	0.06789
MAGED2	MAGE family member D2	Xp11.21	1.527(1.257–1.869)	3.29E-06	1.413(1.089–1.85)	0.00584

## Discussion

The understanding of breast cancer and its strategies for therapy has remarkably improved because of the development of molecular biology in recent years (Redden & Fuhrman, 2013). At present, however, chemo-resistance still poses a major obstacle to satisfactory treatment for breast cancer individuals, with a number of individuals suffering from recurrence and metastasis (Greville et al., 2016; Loibl, Denkert & Von Minckwitz, 2015). In the present study, we conducted WGCNA approach to screen a series of promising indicators for clinical response and survival of HER2-negative BC patients receiving taxane-anthracycline neoadjuvant chemotherapy. Moreover, significantly altered genes and pathways contributing to HER2-negative breast cancer chemo-resistance were also identified.

Compared with previous studies, this is the first network-analyzed WGCNA approach with full thought of high-throughput data including the training dataset of 310 HER2-negative breast cancer samples received taxane-anthracycline treatment and an independent validation set with 198 samples to confirm the relations of the gene modules or hub genes. For module detection of the 5,571 most variant genes, altogether ten modules were identified with a unique color each as an identifier. Module preservation analysis in validating set indicated that the identified modules were reliable as all of the modules’ zsummery statistics were more than 10. Module–clinical trait relationships analysis suggested significant relation of blue module with sensitivity, grade and pam50 while the stage was associated with red module. As module blue also demonstrated significant correlation with DRFS, we selected module blue as the hub module.

Identifying genes related with possible clinical trait is of great interest to elucidate the biological relevant molecular mechanisms. In this study, GO and KEGG enrichment analysis were conducted concerning the genes in hub blue module. Genes in blue module was related with regulation of hormone levels, reproductive system development, response to estradiol, cell growth and mammary gland development according to GO analysis. As for KEGG analysis, genes of blue module demonstrated enrichment in pathways of estrogen signaling, apelin signaling, cAMP signaling, the PPAR signaling pathway and fatty acid metabolism. The identified items of hormone levels, reproductive system development, response to estradiol, estrogen signaling confirmed the indispensable role of hormone regulation in the development as well as the chemotherapeutic response of breast cancer (Folkerd & Dowsett, 2013). Previously, Chen et al., (2012) suggested that PPAR signaling pathway may be a key predictor of breast cancer response to neoadjuvant chemotherapy by results from the microarray data as well as qRT-PCR validation. The role of fatty acid metabolism pathway in HER2-negative breast cancer response with taxane-anthracycline neoadjuvant chemotherapy required further investigations to elucidate.

Traditional clinicopathological and molecular prognostic factors of TNM stage, histological classification, oestrogen and progesterone receptors status failed to effectively assess the benefits of chemotherapy in HER2-negative breast cancer (Prat et al., 2015). Based on high-throughput genomic data, we identified a number of genes which could probably predict response and prognosis for HER2-negative BC patients who received taxane-anthracycline chemotherapy. Ten most relevant genes in blue module (TBC1D9, CA12, ESR1, FOXA1, MLPH, XBP1, AGR2, GATA3, SLC39A6, and MAGED2) were all significantly associated with better DRFS when overexpressed. After further analyzing the relationship between these hub genes and taxane-anthracycline sensitivity, we suggested that all the hub genes significantly associated with neoadjuvant chemotherapy response in the training set, while six genes (CA12, FOXA1, MLPH, XBP1, GATA3 and MAGED2) still accurately predict response in the validating dataset. The expression of carbonic anhydrase XII (CA12) gene which encodes a zinc metalloenzyme participating in acidification of tumor microenvironment, demonstrates correlation with estrogen receptor alpha in human BC. CA12 has previously been reported to be frequently modulated by estrogen through ER alpha in BC cells, which contains a distal estrogen-responsive enhancer region (Barnett et al., 2008). Expression of FOXA1 after neoadjuvant chemotherapy, a forkhead family transcription factor, has been found to be significantly related with distant disease-free survival of stage II or III ER+ HER2- BC patients treated with anthracycline/taxane neoadjuvant chemotherapy (Kawase et al., 2015). XBP1 motivates triple-negative breast cancer via affecting the HIF1 *α* pathway (Chen et al., 2014) and promotes snail expression to induce epithelial-to-mesenchymal transition as well as invasion of breast cancer cells (Li et al., 2015). GATA3 belongs to the GATA family of transcription factors. Aberrant alternation of the reciprocal feedback loop of GATA3- and ZEB2-nucleated repression programs has been found to result in BC metastasis (Si et al., 2015). MAGED2 participates in cell cycle regulation and participates in the process of methionine deprivation which leads to a targetable vulnerability in triple-negative breast cancer cells by promoting Trail receptor-2 expression (Strekalova et al., 2015). The above-mentioned studies indicated that these hub genes might exert specific functions in determining response and prognosis for HER2-negative breast cancer treatment with taxane-anthracycline neoadjuvant chemotherapy and serve as promising biomarkers with potential clinical application in the future, although currently the mechanisms were unclear. The significance and mechanism of the network and core genes in sensitive and prognostic prediction of HER2-negative BC treatment needs further confirmation by large prospective individual cohorts in different ethnicities. Restricted by the unavailability of samples for western blot, it is currently hard for us to detect the protein level of each identified gene, which is a potential limitation of this study. One potential limitation is that the correlation between blue module and sensitivity as well as the red module and the stage were not relatively large, the result of which should therefore be confirmed by future study on the same concern.

In summary, we used WGCNA to suggest a gene network that regulate HER2-negative breast cancer treatment with taxane-anthracycline neoadjuvant chemotherapy, which enriched in pathways of estrogen signaling, apelin signaling, cAMP signaling, the PPAR signaling pathway and fatty acid metabolism. In addition, genes of CA12, FOXA1, MLPH, XBP1, GATA3 and MAGED2 might serve as novel biomarkers predicting chemotherapeutic response and prognosis for HER2-negative breast cancer.

##  Supplemental Information

10.7717/peerj.7515/supp-1 Figure S1 Threshold selection of WGCNA analysis

10.7717/peerj.7515/supp-2 Figure S2 Protein–protein interaction network analysis of the genes in blue module

10.7717/peerj.7515/supp-3 Table S1 Multiple regression of genes in blue modules Adjusted by stage and grade.

10.7717/peerj.7515/supp-4 File S1 Raw data throughout the article analysis process All of the enrichment analysis, all gene in blue module and so on.

## Additional Information and Declarations

### Data Availability

The following information was supplied regarding data availability: Data is available at NCBI GEO under accession numbers GSE25055 and GSE25065.
