# Supplementary figures and images for: Network-based approach to identify biomarkers predicting response and prognosis for HER2-negative breast cancer treatment with taxane-anthracycline neoadjuvant chemotherapy
Source: PeerJ. 2019 Sep 3;7:e7515. doi: 10.7717/peerj.7515 (PMC6730536; doi:10.7717/peerj.7515)

### Scale independence

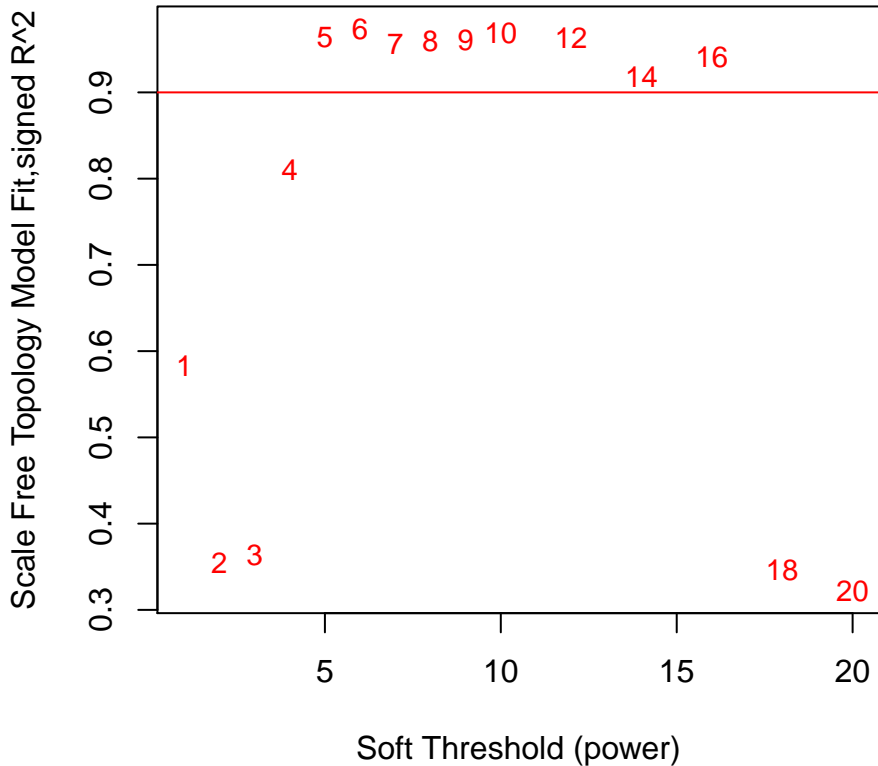

### Mean connectivity

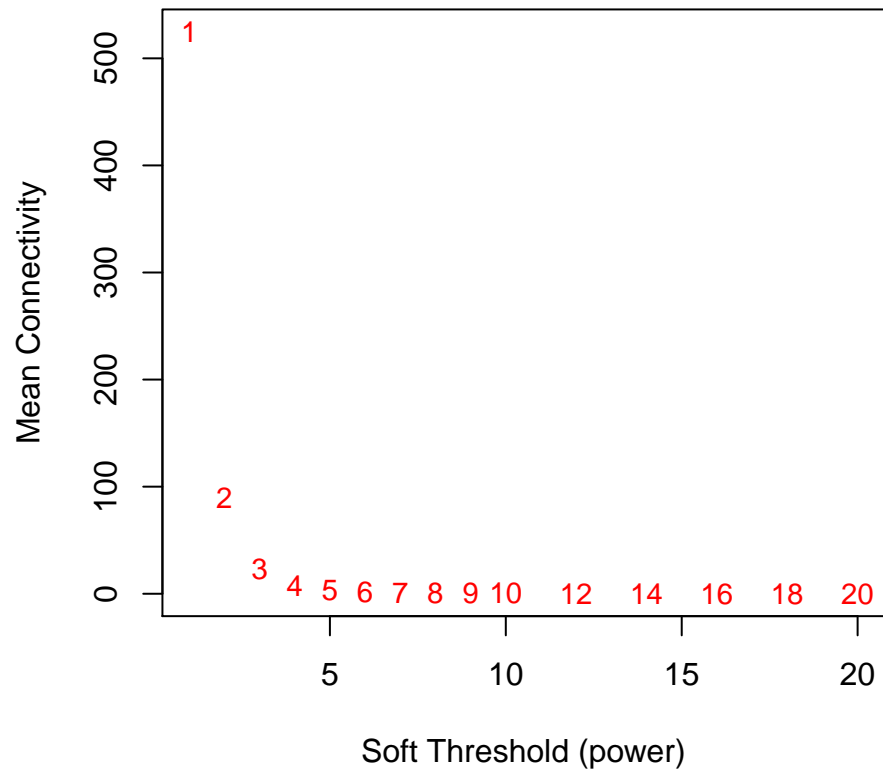

Supplement: Figure S1 [file peerj-07-7515-s001.pdf]

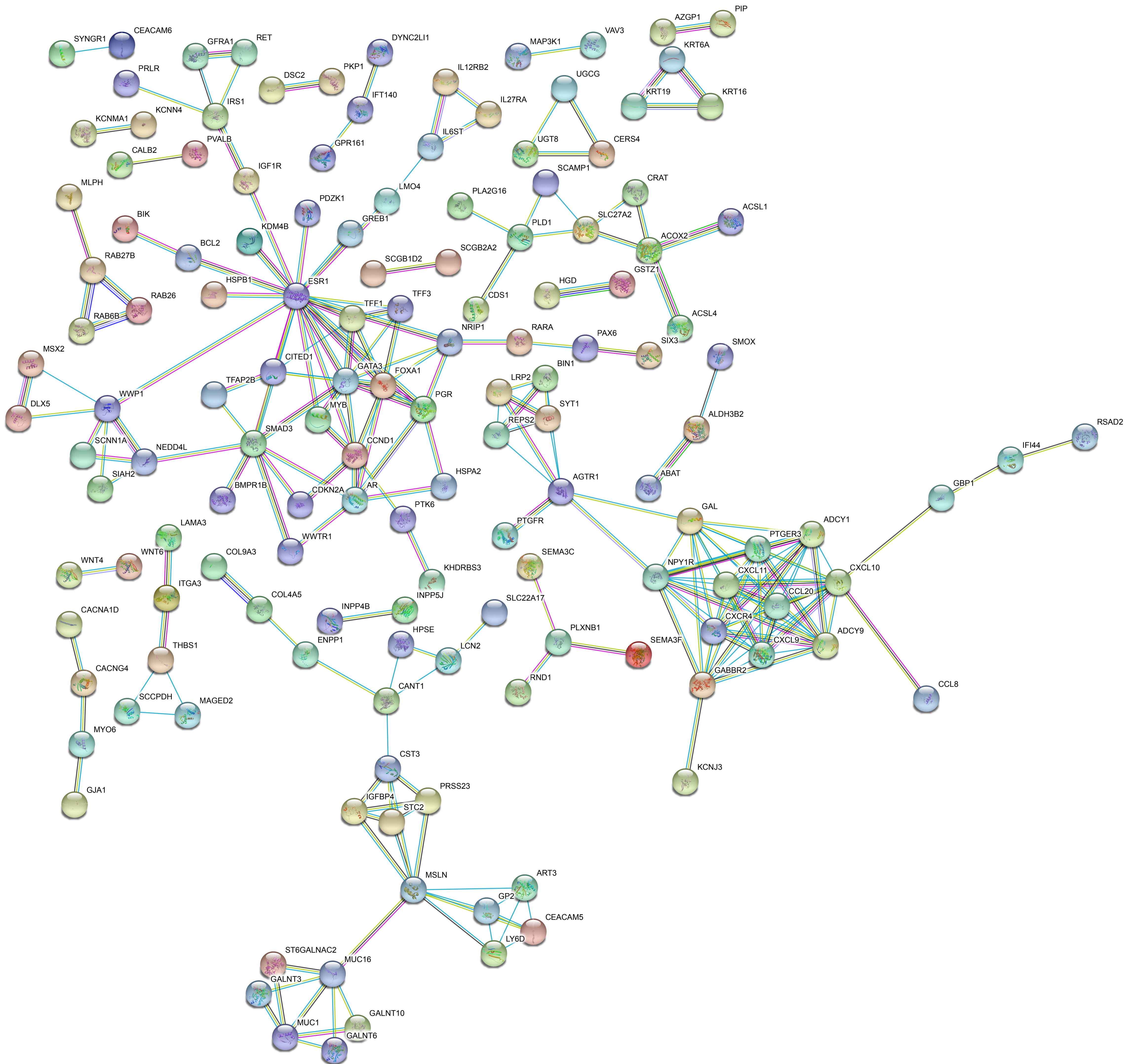

Supplement: Figure S2 [file peerj-07-7515-s002.pdf]
